# Retraction: Effects of GABAB receptor activation on spatial cognitive function and hippocampal neurons in rat models of type 2 diabetes mellitus

**DOI:** 10.1042/BSR-20171184_RET

**Published:** 2020-03-09

**Keywords:** Gamma-aminobutyric acid type B, Type 2 diabetes mellitus, Spatial cognitive function, Hippocampal neurons, Rat models

The authors of the published article ‘Effects of GABAB receptor activation on spatial cognitive function and hippocampal neurons in rat models of type 2 diabetes mellitus’ (*Bioscience Reports* (2018) 38(1); https://doi.org/10.1042/BSR20171184) would like to retract their published article as they have found that the statistical analysis methods used in their study were incorrect, which had led to inaccurate findings. The precise statistical inaccuracies are outlined below:

1. After treatment, before treatment and before execution, the statistical analysis method should be a paired t-test, not an independent sample t-test.
2. A repeated measurement ANOVA rather than a one-way ANOVA should have been used to analyse observations at different time points (1d, 2d, 3d, 4d, 5d, 6d, and 7d).

The authors apologise for any inconvenience caused to the readers and hope to avoid misleading other researchers by retracting their article.

